# Diagnostic value of fetal autopsy after early termination of pregnancy for fetal anomalies

**DOI:** 10.1371/journal.pone.0275674

**Published:** 2022-10-19

**Authors:** Violaine Peyronnet, Olivia Anselem, Laurence Loeuillet, Nathalie Roux, Vassilis Tsatsaris

**Affiliations:** 1 Maternité Louis Mourier, Université de Paris, Assistance Publique- Hôpitaux de Paris, Colombes, France; 2 Maternité Port-Royal, Université Paris Descartes, Groupe hospitalier Cochin Broca Hôtel-Dieu, Assistance Publique-Hôpitaux de Paris, Paris, France; 3 Department of Histology, Embryology and Cytogenetics, Fetal Pathology Unit, Necker-Enfants Malades Hospital, APHP, Paris, France; 4 Université de Paris, Paris, France; Lausanne University Hospital: Centre Hospitalier Universitaire Vaudois (CH), SWITZERLAND

## Abstract

**Background:**

In early terminations of pregnancy for fetal anomaly (TOPFA) without identified cytogenetic abnormality, a fetal autopsy is recommended for diagnostic purposes, to guide genetic counseling. Medical induction, which allows analysis of a complete fetus, is generally preferred over surgical vacuum aspiration. Our objective was to assess the diagnostic value of fetal autopsies in these early terminations, relative to the first-trimester ultrasound, overall and by termination method.

**Materials:**

For this retrospective study at the Port Royal Maternity Hospital, we identified all TOPFA performed from 11 weeks to 16 weeks diagnosed at the first-trimester ultrasound in cases with a normal karyotype. The principal endpoint was the additional value of the autopsy over /compared to the ultrasound and its impact on genetic counseling, globally and by termination method. The secondary objective was to compare the complication rate by method of termination.

**Results:**

The study included 79 women during period of 2013–2017: 42 with terminations by medical induction and 37 by aspiration. Fetal autopsy found additional abnormalities in 54.4% of cases, more frequently after medical induction (77.5%) than after aspiration (21.4%, p < .01). Genetic counseling was modified in 20.6% of cases, more often after induction (32.5% vs 3.6%, p < .01). The length of stay was significantly longer and a secondary aspiration was required in 16,7% of case in the medical induction group (p < .01).

**Conclusion:**

Medically induced vaginal expulsion appears preferable and can change genetic counseling for subsequent pregnancies.

## Introduction

According to French law, a fetal anomaly can lead to a termination of pregnancy (TOP) if the couple so requests, after an opinion by a prenatal diagnostic center that there is "a strong probability that the child to be born is affected by a particularly severe condition recognized as incurable at the time of diagnosis." Two techniques can be used for early TOP before 16 weeks: either surgical vacuum aspiration, or medically induced vaginal expulsion. Surgical aspiration has the advantage of being a rapid procedure, most often performed́ on an outpatient basis. Its disadvantage is that it does not preserve the whole fetus; the resulting fragmentation makes a fetal autopsy difficult and potentially of little value. Moreover, like all surgery, it involves risks, especially that of uterine perforation. Medical induction of vaginal expulsion has the advantage of preserving the bodily integrity of the fetus, but the procedure is longer and may be more psychologically traumatic for the woman [1, 2]. It also involves the risk of trophoblastic retention and thus of a secondary aspiration. For these reasons, surgical aspiration is often preferred when cytogenetic examinations before TOP have established the etiologic diagnosis, so that a fetal autopsy for this purpose is unnecessary. This is the case for aneuploidies and for chromosomal imbalances identified by array-based comparative genomic hybridization (aCGH). When no etiologic diagnosis is available before the TOP, medically induced vaginal expulsion is systematically proposed to enable the performance of a fetal autopsy. The mother or the couple receives medical information about the advantages and disadvantages of each method and makes the final decision about the procedure and about the autopsy. Requests for autopsies have diminished over the past several years [3], associated with cultural factors or religious beliefs, but also because of the simplicity of the vacuum aspiration procedure.

Ultrasound does not have a 100% sensitivity rate for screening early anomalies [4], nor is its specificity for diagnosis 100%. Most authors [5] and teams therefore advise a systematic fetal autopsy after TOP to identify additional abnormalities, guide possible subsequent genetic exploration, and refine genetic counseling for future pregnancies. The diagnostic value of fetal autopsies after 18 weeks has been studied́ extensively [3, 6–11]; much less is known about the value of earlier autopsies [12]. In 2011, we examined early TOPFA before 16 weeks in our department [12] and showed that genetic counseling benefited from TOP by medically induced vaginal expulsion. In view of the technological advances in ultrasound and in fetopathology, we decided to continue this research.

We therefore examined the value of fetal autopsy in case of early TOPFA before 16 weeks in cases without any fetal cytogenetic abnormality. The principal objective of the study was to assess the diagnostic value of an autopsy over prenatal ultrasound and its impact on genetic counselling, overall and by method of termination: surgical aspiration or medically induced vaginal expulsion. The secondary objective was to compare the complication rate by method of termination.

## Materials and methods

### Design-case selection

This retrospective observational study took place at the Port Royal Maternity Hospital, which has both a prenatal diagnosis department and a multidisciplinary prenatal diagnostic center. The study reviewed records for the calendar years 2013 through 2017. To assess the diagnostic utility of fetal autopsies, we included women with a fetal anomaly diagnosed at the first-trimester ultrasound that resulted in a TOPFA before 16 weeks of gestation. We selected all TOPFA performed from 11 weeks to 16 weeks because it allowed us to include the TOP performed for anomalies screened during the first-trimester ultrasound, while leaving time for women to think their decision through thoroughly and to schedule and complete the TOPFA.

Exclusion criteria were TOP for a maternal indication or for genetic or chromosomal abnormalities (e.g., aneuploidy and pathogenic imbalances identified by karyotype or array-based comparative genomic hybridization (aCGH)) diagnosed before TOP. For the analysis of main outcome, we further excluded cases where no autopsy was performed.

All women underwent a first-trimester ultrasound examination between 11 weeks and 13 weeks+6 days. The pregnancy was dated by measuring the crown-rump length at this examination. After the identification of an anomaly during this ultrasound, a second ultrasound scan was routinely performed by an expert ultrasonographer to confirm the anomaly and search for additional associated abnormalities. Depending on the abnormalities found, a trophoblast biopsy for karyotype analysis by direct examination and aCGH (with a resolution of 1Mb) was proposed. The time to obtain the results was 3 days for the karyotype and about 15 days for the CGH. There was no Fluorescent In Situ Hybridization (FISH) performed.

After the request for TOPFA, in the absence of an etiological chromosomal or genetic diagnosis, a medically induced vaginal expulsion followed by a fetal autopsy was recommended, but the final choice for the method of termination belonged to the woman. An autopsy of the products of conception was nonetheless routinely suggested for the women choosing vacuum aspiration for TOPFA. For women who did not want a fetal autopsy, external examination of the fetus and radiography could be performed.

### Protocol for methods of termination

Whatever the method, given the term, fetal demise was not induced prior to the procedure. The protocol used for each procedure was identical between the gestational ages of 11 weeks of gestation (WG) and 16 WG.

#### Method for termination of pregnancy by aspiration

Women who chose aspiration for early TOP (before 16 weeks) received 200 mg of oral mifepristone two days before the procedure. The aspiration was performed on an outpatient basis. The morning of the procedure, the woman received 400 μg of misoprostol vaginally, 2 hours before the aspiration. The procedure took place under general anesthesia in the operating room. Mechanical cervical dilatation was performed with a Hegar dilator set; the aspiration was then performed with a rigid cannula and, if necessary, Winter forceps. Dilatation and aspiration were performed under ultrasound control with systematic verification that the uterus was empty at the end of the procedure. Most of these fetuses were fragmented.

#### Method for termination of pregnancy by induction of labor

Women who choose medically induced vaginal expulsion also received 200 mg of mifepristone 2 days before the procedure. They were hospitalized the morning of the induction. In the delivery room they received misoprostol, administered́ vaginally, 400 μg every three hours, under epidural anesthesia. After the expulsion, the medical team verified the placental delivery and performed systematically a manual uterine examination, routinely followed by ultrasound confirmation that the uterus was empty. A secondary aspiration was performed if indicated. Most of these fetuses are intact.

### Protocol for the fetal autopsy

After the mother or couple consented in writing, the autopsy was performed́ according to a standardized protocol. The products of conception or the fetus were transported in a fresh state to the laboratory in the hours after the procedure.

#### Pathologic sampling of specimens obtained by aspiration

For TOP by aspiration, the products of conception were washed, fixed in formalin, and analyzed. The macroscopic examination consisted of various fetal and extraembryonic structures (fetal organs, placenta, and umbilical cord). The identifiable items underwent a morphologic examination with photographs and radiography. A histologic analysis was then performed on all the elements collected.

#### Pathologic sampling of specimens obtained by induction of labor

A fetus expelled after medical induction was first weighed́ and measured, then examined externally, photographed, and radiographed. The organs were then separated and examined whole. Two final macroscopic examinations followed one internal, of the viscera, to search for malformations, and one neuropathological. Then all the organs were removed and analyzed histologically. Finally, the placental, membranes, and umbilical cord were analyzed macroscopically and then histologically.

All fetal autopsies throughout the study period were performed́ by one of only two different specialists.

### Variables collected and variables of interest

We collected the women’s characteristics, the ultrasound, autopsy, and cytogenetic data, as well as the contents of the follow-up visits, including the delivery of the post-TOPFA results, especially the genetic consultations. The data about the TOPFA, its indication, gestational age at performance, method of termination, any complications, and length of hospital stay were also collected. The indications for TOPFA were grouped in several categories: cerebral abnormalities, comprising all head malformations, including exencephaly, bone abnormalities, hygromas, isolated increased nuchal translucency, abdominal wall defects, neural tube defects, cardiac, lumbosacral, and other abnormalities, and multiple malformation syndromes.

As required by French law and regulations, this study was approved by the national data protection authority (Commission Nationale de l’Informatique et des Libertés, CNIL n° 1755849) and by the appropriate ethics committees, i.e. the advisory committee on the treatment of personal health data for research purposes (CCTIRS: Comité Consultatif sur le Traitement de l’Information en matière de Recherche, approval granted November 18, 2010; reference number 10.626). Women were informed that their records could be used for the evaluation of medical practices and were provided the option to opt out of these studies. All data were anonymized before the analysis.

#### Endpoints

The principal endpoint was the diagnostic value of the fetal autopsy, compared with̀ the ultrasound, defined by the demonstration of at least one supplementary abnormality (major or minor) not detected by ultrasound. This analysis was conducted for all cases that had autopsies and by method of termination.

We also sought to assess whether the autopsy results modified the genetic counseling: we considered that it did so if the additional abnormalities that the autopsy identified or did not exclude, guided the diagnosis toward a specific etiology and provided specific information for the follow-up of subsequent pregnancies. This information could concern the risk of recurrence or indicate a potential diagnosis to be tested in subsequent pregnancies by an invasive sample or a specific ultrasound follow-up.

At the time of the study, there was no whole genome done in a systematic way. Only targeted genetic research was carried out according to the anomalies found on ultrasound and autopsy.

When the fetal autopsy concluded in favor of an anomaly of sporadic onset without any indication for a specific follow-up for subsequent pregnancies, only ultrasounds for reassurance were recommended. We chose not to categorize these situations as modifications of genetic counseling as such, especially as they could have been implemented in some cases based only on the conclusions of the ultrasound.

In order to limit the potential confusion bias linked to the gestational age (14–16 weeks) at which the TOPFA is performed, we carried out a sensitivity analysis by evaluating the same endpoints on a population including only pregnancy terminated < = 14 weeks.

Secondary analyses for these same outcome measures were conducted in the subgroup with cerebral abnormalities.

We also compared the complication rates according to the method of termination: the performance of a secondary aspiration, maternal hemorrhage (blood loss greater than 500 ml), intrauterine retention (with an anteroposterior diameter more than 15 mm), or infection (that required antibiotic treatment for suspected postpartum endometritis). We also compared length of stay by method of termination.

### Statistical analysis

We first described our population by their means (and standard deviations, SDs) for the continuous variables and by percentages for the categorical variables. The categorical variables of interest were then compared either by Chi-2 or Fisher’s exact tests. Student’s t test was used to analyze the quantitative variables. All statistical analyses were performed with Stata 16 software. Differences were considered significant when p< 0.05.

## Results

### Study population and analysis

During the 5-year period of 2013–2017, 318 TOP took place before 16 weeks of gestation at our prenatal diagnosis center. We excluded 239 women: 190 with TOP for cytogenetic abnormality: 102 trisomy 21, 42 trisomy 18, 17 trisomy 13, 14 monosomy X (Turner syndrome), 7 triploidy, 9 other aCGH abnormalities, 20 TOP for genetic diseases, and 29 for maternal disease (e.g., cancer, exposure to teratogenic treatments (without fetal anomalies in our study), preterm premature of membranes).

**Fig 1:**
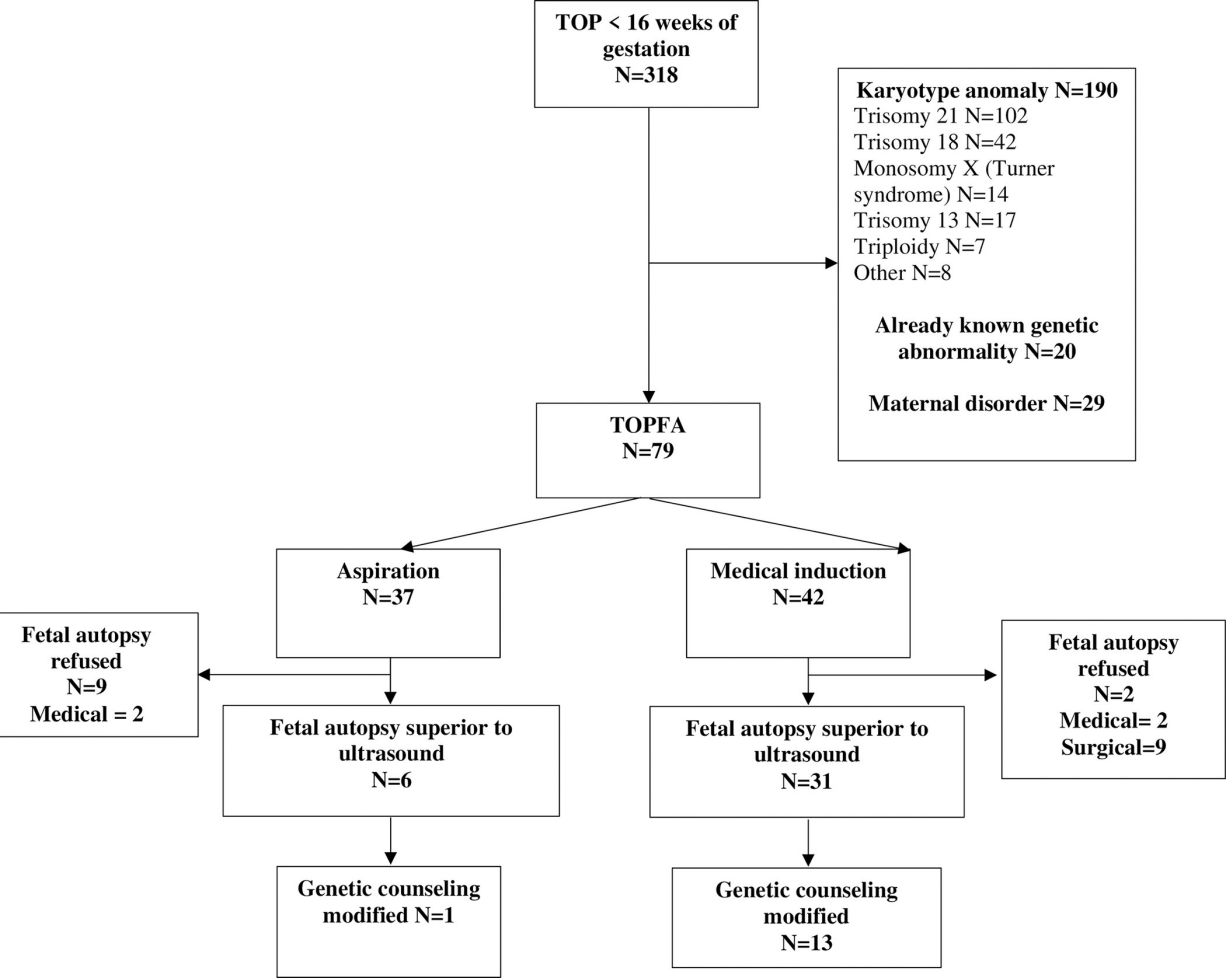
Study population and analysis. TOPFA: termination of pregnancy for fetal anomaly, weeks: weeks of gestation, Medical: medical induction, Surgical: vacuum aspiration.

We therefore included 79 women: 53.2% (N = 42) had TOPFA by medical induction, and 46.8% (N = 37) had TOPFA by aspiration. Among the 42 women who underwent medical induction, two wanted only an external examination without an autopsy, and among the 37 women with aspiration, 9 did not consent to a fetal autopsy (**Fig 1**).

### Description of the population and their terminations of pregnancy for fetal anomalies

Their mean age was 32.3 years (+/-5 years), and 48% (38/79) were nulliparous. Maternal characteristics did not differ significantly between the medical induction and aspiration groups (**Table 1**). The most frequent indication for TOPFA was a cerebral abnormality (34.2%), and then multiple congenital malformations (27.8%).

**Table 1:** Characteristics of the mothers and the terminations of pregnancy.

Characteristics	All termination of pregnancy N = 79	Medical induction N = 42	Aspiration N = 37	*p*
**Age (years), (mean ± SD)**	32.3 (5.0)	32 (4.6)	32.7 (5.5)	.5
**Nulliparous** (%, *n*)	48.1 (38)	42.9 (18)	54 (20)	.3
**GA at 1**^**st**^ **consultation** *(mean*, *+/-SD)*	12.3 (1.3)	12.4 (1.3)	12.3 (0.9)	.7
**Mother’s place of birth** (%, *n*)				.6
France	64.1 (50)	56.1 (23)	73 (27)	
North Africa	10.3 (8)	12.2 (5)	8.1 (3)	
Sub-Saharan Africa	9 (7)	12.2 (5)	5.4 (2)	
Asia	6.4 (5)	9.8 (4)	2.7 (1)	
French overseas departments and territories	2.6 (2)	2.4 (1)	2.7 (1)	
Other European countries	7.7 (6)	7.3 (3)	8.1 (3)	
**Consanguinity** *(%*, *n*)	1.3 (1)	0	1 (2.7)	.3
**ART** *(%*, *n*)	5.1 (4)	2.4 (1)	8.3 (3)	.2
**Indication for TOPFA** *(%*, n)				.07
Cerebral	34.2 (27)	21.4 (9)	48.6 (18)	
Bone	7.6 (6)	9.5 (4)	5.4 (2)	
Hygroma isolated	16.5 (13)	14.3 (6)	18.9 (7)	
Wall closure	7.6 (6)	7.1 (3)	8.1 (3)	
Neural tube closures	3.8 (3)	7.1 (3)	0	
Cardiac	2.5 (2)	4.8 (2)	0	
Multiple malformation syndrome or other	27.8 (22)	35.7 (15)	18.9 (7)	
**Trophoblast biopsy before TOPFA** *(%*, n*)*	41.8 (33)	61.9 (26)	18.9 (7)	< .001
**GA at TOPFA, weeks** *(mean +/-SD)*	14.0 (1.1)	14.6 (1.0)	13.3 (0.9)	< .001
**TOP < = 14 weeks** *(%*, *n)*	53.2 (42)	28.6 (12)	81.1 (30)	< .001
**TOP>14 weeks** *(%*, *n)*	46.8 (37)	71.4 (30)	18.9 (7)	

ART: Assisted reproduction, GA: Gestational age, SD: Standard deviation, TOPFA: Termination of pregnancy for fetal anomaly

The mean gestational age at TOPFA was 14 weeks (+/-1.1). Medical inductions took place later than aspirations: 14.6 weeks versus 13.3 weeks (*p* < .001). 53.2% (42/79) were performed before or at 14 weeks, 28.6% (12/42) in the medical inductions and 81.1% (30/37) in the aspirations.

41.8% (33/79) had a trophoblast biopsy, 61.9% (26/42) in the medical inductions and 18.9% (7/37) in the aspirations.

### Value of fetal autopsy (Table 2)

**Table 2:** Value of fetal autopsy, globally and by method of termination.

	All termination of pregnancy N = 79	Medical induction N = 42*	Aspiration N = 37**	*p*
**Autopsies performed** *(n*, *%)*	68/79 (86.1%)	40/42 (95.2%)	28/37 (75.7%)	.03
**Diagnostic value compared with ultrasound** *(n*, *%)*	37/68 (54.4%)	31/40 (77.5%)	6/28 (21.4%)	< .001
**Modification of genetic counseling** *(n*, *%)*	14/68 (20.6%)	13/40 (32.5%)	1/28 (3.6%)	< .01

*2 Refusal of complete autopsy

**9 Refusal of complete autopsy

#### Principal endpoint

Among the fetal autopsies (N = 68), 54.4% (37/68) provided more information than ultrasound; this was the case more frequently in the medical inductions (77.5%, 31/40) than in the aspirations (21.4%, 6/28) (*p* < .001) (**Table 2**). For example, the autopsy enabled the identification of signs associated with what had appeared to be an isolated hygroma on ultrasound (n = 4) and the diagnosis of amniotic band syndrome with multiple malformation syndromes (n = 3) and with a short umbilical cord (n = 1). It also made it possible to diagnose Cantrell’s pentalogy in two cases, although the medium intermediate celosomia had appeared isolated on ultrasound.

The fetal autopsy led to a change in the genetic counseling provided in 20.6% of cases (14/68), again, more often in the medical induction group (32.5%, 13/40) than in the aspiration group (3.6%, 1/28) (*p* < .001) (**Table 2**). The cases for which fetal autopsies modified the genetic counseling are detailed in **Table 3.**

**Table 3:** Modifications of genetic counseling after fetal autopsy following early TOPFA.

Case	TOPFA method	Ultrasound signs	Fetal autopsy	Genetic counseling
**1**	E	MMC	MMC, renal cysts	Low risk of recurrence
**2**	E	Ventricular dilation	Ventricular dilation, hemorrhagic remodeling, atresia of the aqueduct of Sylvius, probable hemorrhagic stroke	Test for platelet incompatibility
**3**	A	Short, rod-shaped LL, hygroma, enlarged bladder, spinal abnormalities, SGA	Severe micromelia, 4 limbs with deformed long bones, left thumb in abduction. Normal cartilage. Bifid metacarpals, dilated bladder, dysplastic kidneys	Risk of recurrence, possible association. Couple lost to follow-up.
**4**	E	Agenesis of forearm bones	Bilateral renal agenesis, colon to bladder outlet, imperforate anus, right radial club hand, with agenesis of thumb, agenesis of radius, dysmorphism	Transmitted as an autosomal dominant trait mutation SALL 1 or 4
**5**	E	Hygroma	Dysmorphism: hypertelorism, small low-set ears, cervical hygroma, horseshoe kidneys	Possible syndromic association with risk of recurrence. Couple lost to follow-up
**6**	E	Hygroma	Hepatic calcifications, cardiac valve alignment	Heart disease. Fetal cardiac ultrasound
**7**	E	Posterior encephalocele, rachischisis, misplaced hands, SGA	Amniotic band disease with craniocervical rachischisis, MMC, clubhand, syndactyly, digital amputation, visible bands. Cleft palate, endocardial fibroelastosis	Accidental for amniotic bands but supplementary genetic exploration for cleft and fibroelastosis with request for normal maternal cardiac ultrasound
**8**	E	Hygroma	Facial dysmorphism: hypertelorism, oblique palpebral fissures, low-set posteriorly rotated ears. Cervical cystic hygroma, SUA. Heart: bifid apex, hypoplastic right heart syndrome, tricuspid valve dysplasia, disorganization of the right ventricular myocardium	RASopathy on genetic testing (BRAF gene variant), mother not a carrier, husband did not want to be tested
**9**	E	Hygroma	Dysmorphism suggestive of Noonan syndrome, generalized subcutaneous edema of the pterygium colli. Signs of tissue hypoxia	RASopathie with de novo mutation c.7770A>G BRAF gene implicated in a craniofaciocutaneous syndrome
**10**	E	Short limbs and ribs	Postaxial polydactyly of the 4 extremities, left foot syndactyly. Cranial hypoplasia of the skull bones, facial anomaly, posterior cleft palate, tongue anomaly. Short ribs, very short long bones, hypomineralization, round shoulder blades, small spiculated iliac wings. SUA.	Short rib polydactyly syndrome, type 7 WDR35 gene Potential autosomal recessive transmission.
**11**	E	Major ventricular dilation	Vertebral body abnormalities, hydrocephaly, rhombencephalosynapsis, a complex heart defect, right juxtaposition of the atrial appendages, transposition of the great vessels, aortic dilation, severe pulmonary stenosis, VSD, Meckel diverticulum	probable VACTERL-H Possible autosomal recessive transmission Gene not found Monthly ultrasound follow-up
**12**	E	Abnormalities of the lower limbs, upper limb amelia	Bilateral phocomelia, short thumbs, clinodactyly 5th. Radiography: suspected right tibial curvature and hypoplastic left fibula	Thrombocytopenia-absent radius (TAR) syndrome Autosomal recessive transmission Propose early chorionic villous sampling in subsequent pregnancy because both parents carry the hypomorphic RBM8A allele
**13**	E	Megabladder, urethral recess	Multiple malformation syndrome, megabladder, urethral atresia with bilateral renal repercussions, vertebral bodies not visible in cervical vertebrae, abnormalities of external genitalia, anorectal malformation, (rectal atresia and fistula), uterovaginal aplasia with rudimentary tubes	MURCS syndrome Transmitted as an autosomal dominant trait with incomplete penetrance and variable expressivity
**14**	E	Cystic hygroma, long thumbs, mild varus foot, deviated cardiac axis	Fetal hydrops, subcutaneous edema, bilateral pleural effusion, posterior cleft palate, thymic agenesis, conotruncal heart defect	Di Georges syndrome Parental samples normal, therefore de novo. Low risk of germline mosaicism, amniocentesis possible.

TOPFA: Termination of pregnancy for fetal anomaly, E: vaginal expulsion after medical induction, A: surgical vacuum aspiration, MMC: myelomeningocele, LL: lower limbs, SGA: small-for-gestational age, SUA: single umbilical artery, RV: right ventricle, VSD: ventricular septal defect

#### Sensitivity analysis: TOPFA < = 14 weeks Table 4

When restricting to TOPFA performed before or at 14 weeks, 42 pregnancies were included.

**Table 4:** Value of fetal autopsy, globally and by method of termination for TOPFA< = 14SA (sensitivity analysis).

	All termination of pregnancy N = 42	Medical induction N = 12*	Aspiration N = 30**	*p*
**Autopsies performed** *(n*, *%)*	35/42 (83.3%)	11/12 (91,7%)	24/30 (80%)	.6
**Diagnostic value compared with ultrasound** *(n*, *%)*	15/35 (42.9%)	10/11 (90.9%)	5/24 (20.8%)	< .01
**Modification of genetic counseling** *(n*, *%)*	4/35 (11.4%)	3/11 (27.3%)	1/24 (4.2%)	.046

*1 Refusal of complete autopsy

**6 Refusal of complete autopsy

Among the fetal autopsies (N = 35), 42.9% (15/35) provided more information than ultrasound; this was the case more frequently in the medical inductions (90.9%, 10/11) than in the aspirations (20.8%, 5/24) (*p* < .01) (**Table 4**).

In this sensitivity analysis, the fetal autopsy led to a change in the genetic counseling provided in 11.4% of all cases (4/35), and this was still significantly more often in the medical induction group (27.3%, 3/11) than in the aspiration group (4.2%, 1/24) (*p* = .046).

#### Subgroup analyses: Cerebral abnormalities (Table 5)

Analysis of the subgroup with cerebral abnormalities (N = 27) showed that the autopsy had a greater diagnostic value than ultrasound in 40% of the cases (8/20), again more frequently in case of medical induction group (71.4%, 5/7) than in aspirations (23.1%, 3/13) (*p* = .03) (**Table 5**). For example, in a case of ultrasound-suspected exencephaly, the autopsy identified a multiple malformation syndrome with iniencephaly, occipital meningocele, cervicodorsal rachischisis, external genitalia abnormalities, and pulmonary hypoplasia. In other cases, the autopsy identified vertebral, costal, and renal abnormalities associated with exencephaly. In two cases, it enabled a diagnosis of amniotic band syndrome. In one case of hydrocephaly, it identified signs suggesting a hemorrhagic stroke.

**Table 5:** Value of fetal autopsy globally and by method of termination for cerebral abnormalities.

	All termination of pregnancy N = 27	Medical induction N = 9*	Aspiration N = 18**	*p*
**Autopsies performed** *(n*, *%)*	20/27 (74.1%)	7/9 (77.8%)	13/18 (72.2%)	0.3
**Diagnostic utility compared with ultrasound** *(n*, *%)*	8/20 (40%)	5/7 (71.4%)	3/13 (23.1%)	.03
**Modified genetic counseling** *(n*, *%)*	2/20 (10%)	2/7 (28.6%)	0/13 (0%)	.04

*2 Refusal of complete autopsy

**5 Refusal of complete autopsy

### Maternal complications according to method of termination

As the different protocols suggested, the length of stay was significantly longer in the medical induction group (2.1 days +/-0.5) than in the aspiration group (1 day +/-0.2, *p* < .001). In 16.7% of the medical inductions, a secondary aspiration was required for retention of the products of concept (N = 6) or hemorrhage (N = 1); there was none in the aspiration group (*p* < .001).

Post-expulsion retention was observed in 11.4% of all cases (n = 9): 14.3% (6/42) in the medical induction group and 8.1% (3/37) in the aspiration group, *p* = .5. There was one case of hemorrhage in each group and one infection in the medical induction group (**Table 6**).

**Table 6:** Maternal complications globally and by method of termination.

Complications	All termination of pregnancy N = 79	Medical induction N = 42	Aspiration N = 37	*p*
**Length of stay, day** *(mean +/-SD)*	1.6 (0.6)	2.1 (0.5)	1.0 (0.2)	< .001
**Secondary aspiration** *(%*, *n)*	8.9 (7)	16.7% (7)	0 (0)	< .001
**Intrauterine retention** *(%*, *n)*	11.4 (9)	14.3 (6)	8.1 (3)	.5
**Hemorrhage** *(%*, *n)*	2.5 (2)	2.4 (1)	2.7 (1)	1
**Infections** *(%*, *n)*	1.3 (1)	2.4 (1)	0 (0)	1

Mean: mean +/-SD: standard deviation

## Discussion

This study showed that in case of medical TOP without cytogenetic abnormality performed after the diagnosis of a fetal anomaly at the first-trimester ultrasound, the fetal autopsy provided additional information over and above that of the ultrasound in 54.4% of the cases, and more frequently after a medical induction (77.5%) than after a vacuum aspiration (21.4%, *p* < .001). It led to an important change in the genetic counseling and management for the subsequent pregnancy in 20.6% of cases, 32.5% of those after medical induction and 3.6% after aspiration; the difference between the two groups was statistically significant (*p* < .001).

The principal strengths of our study lie in the performance of fetal autopsies by two physicians specialized in fetal pathology, according to a standardized examination protocol for each method of termination. The conclusions were systematically discussed at a multidisciplinary meeting. We used a strict definition of modification of genetic counseling. Moreover, before each decision for TOPFA, the ultrasound was repeated for independent verification by a specialist. These cases were collected over a limited period of 5 years that allows us to consider that their management was homogeneous. Our collection of complications was exhaustive because the women were all seen at follow-up visits after their TOPFA, and secondary complications were always collected.

The limitation due to its retrospective nature and the limited size of our population did not allow any subgroup analysis of the anomalies. Some bias might have been present, especially related to the choices of procedure according to gestational age. Thus, a study of the characteristics of the TOP shows that those performed by aspiration took place at a significantly earlier gestational age than those by medical induction. This might constitute a confounding factor for the assessment of the value of autopsies after medical induction. Nonetheless, the absolute value of the difference in gestational age is low and we performed a sensitivity analysis on TOPFA performed before or at 14 weeks and the results were similar and significant. Moreover, it is important to note that reasons for TOPFA did not differ between the groups.

These results point in the same direction as those of our team’s first study on this topic between 2006 and 2008 [12]. That study reported that the autopsies were diagnostically valuable in 42% of the cases, with a greater benefit in the medical inductions, albeit without a statistically significant difference (65% in the medical induction group versus 20% in the aspiration group). It also found that genetic counseling was modified in 37% of the cases, with a difference between the groups: 62% in the medical induction group versus 13% in the aspiration group, *p* < .01. The difference in the rate observed in our study for the modification of genetic counseling can be explained by our more restrictive definition; we did not include cases in which the autopsy resulted in a conclusion that the abnormality was probably sporadic. We decided not to combine the results of the two studies for the analyses because there were notable improvements between the two periods in the screening and diagnosis of malformations by ultrasound improvement in the ultrasound instruments.

Our finding that autopsies modified genetic counseling, regardless of method of termination, are similar to those about for TOP after 18 weeks [3, 6–11]. Those studies reported that diagnostic benefits occurred in 18 to 51% of cases [12]. A systematic review of the literature published in 2017 by Rossi et al., including results from 19 studies and 3,534 fetal autopsies, found that autopsy and ultrasound results were equivalent in 68% of the cases [11]. In 22.5%, they found additional abnormalities, and in 3.8% of the cases, these modified the diagnosis. These percentages are a little lower than ours. Nonetheless, the endpoint was different since we chose the value of the additional signs from the fetal autopsy, compared with a first-trimester ultrasound alone. Moreover, in this review of the literature, the mean gestational age at which the abnormalities were detected on ultrasound ranged between 17 and 20 weeks, while our study considered terminations performed for abnormalities observed during the first-trimester ultrasound, that is, before 14 weeks. We might wonder if the contribution of the autopsy differs by the gestational age at which the TOPFA is performed, especially if it is still earlier in pregnancy. That is, even if an autopsy is possible at 11 weeks, at this stage of morphogenesis and histogenesis, some abnormalities may not yet be able to be diagnosed. Nonetheless this problem also occurs with ultrasound, some abnormalities are not visible at an early gestational age.

The fetal autopsy modified the genetic counseling results in 20.6% of cases, by making it possible to enable prenatal diagnosis for the next pregnancy: by conducting a work-up for the couple, early ultrasound scans if there is a risk of recurrence, possible karyotyping or a genetic examination. Actually, the autopsy provided relevant information even when it did not find additional abnormalities compared with ultrasound. That is, it allowed a conclusion, for example, that the anomaly was indeed isolated and not syndromic, and thus probably sporadic. There is thus no indication for a prenatal diagnosis for a subsequent pregnancy. The benefit provided by fetal autopsy in our study would perhaps be now greater because the fetal autopsy leads to proposal for exome or genome if there is a suspicion of genetic pathology.

Nonetheless, even without modifying the genetic counseling, the management of the next pregnancy was often modified in practice, with the introduction of specifically focused ultrasounds. Another example, folic acid at a dose of 5 mg was proposed́ for subsequent pregnancies for women with neural tube defects. These modifications were not necessarily due to an additional finding from the autopsy but could have been proposed́ based on the abnormalities found on the ultrasound alone. We therefore did not consider in these situations that the autopsy modified the genetic counseling. In two cases, the autopsy added the finding of a myelomeningocele to multiple malformation syndromes already visualized on ultrasound; it thus justified folic acid supplementation at a dose of 5 mg for subsequent pregnancies.

We conducted a secondary analysis in the subgroup of cephalic abnormalities, for these diagnoses might appear to be modified by an autopsy only rarely. If this was confirmed, vacuum aspiration could be performed in most cases. Our results showed that autopsy has substantial value in a substantial portion of these cases (40%), with once again a difference between the methods of termination.

A difference in the value of the autopsy analysis by the method of termination also arises for bone abnormalities where the contribution of the bone radiographs that can be performed on the product of aspiration could lead to a conclusion that this method of termination would be "sufficient". Larger complementary studies focused on some types of abnormalities identified early could be interesting to optimize the discussion with patients about the method to prefer.

We found more complications (secondary aspiration and retention) after medical induction than after aspiration and a longer hospital stay. The results by Gitz et al. [12] were similar. We note that the absolute number of complications were few, and they were minor. One of the limitations of our study is the small number of patients available for comparing complication rates (retention, hemorrhage) by method of termination. Other studies compared́ these different methods [13, 14]. Thus Lohr et al. reviewed the literature and showed fewer and milder undesirable effects after aspiration than after medical induction (OR 0.06, 95% CI 0.01–0.76), which was accompanied by more pain. Nonetheless, acceptability and efficacy were finally identical in the two groups [13].

One of the important questions remains women’s experiences and psychological status after TOP. That is, the benefit provided by the autopsy after medical induction must be balanced with the potential benefits associated with the simplicity of the aspiration procedure. The unavailability of a reliable and exact diagnosis after TOP or the lack of information can also have an important impact on the women’s psychological prognosis. Accordingly, Korenromp et al. [15] assessed women’s psychological status at 4, 8, and 16 months after their termination. They observed that 46% had posttraumatic stress at 4 months and 20.5% at 16 months. Factors predictive of PTSD were: a high level of doubt at the time of the decision, lack of support from their partner, higher gestational age, and religious convictions. Other studies have compared both methods of termination according to the women’s perceptions and showed that the surgical pathway is more acceptable [1, 2]. Thus Kelly et al. reported that after aspiration, 100% of the women would prefer to undergo the same procedure if they had to have another TOP, compared with 53% in the group of medical induction (*p* < .001). Among the women undergoing aspiration, not one found the procedure more traumatizing than expected, compared with 53% after medical induction (*p* = .001), which involved more bleeding (*p* = .003) and more pain (*p* = .008).

## Conclusion

For early TOPFA before 16 weeks, medical induction with vaginal expulsion seems preferable because it enables a more complete and exact analysis of a whole fetus and is more likely to modify genetic counseling or the management of subsequent pregnancies. The balance between the need for a precise diagnosis, the woman’s psychological status, and the potential obstetric complications must be systematically discussed before TOPFA. The benefits and risks of the different procedures must be clearly explained so that women can make an informed choice.

Additional studies to analyze the value of fetal autopsies by gestational age at the time of TOP and by type of abnormality might be interesting. Prospective studies would be necessary to assess the physical and psychological consequences of the different methods.
